# Cubonavicular Coalition in a Pediatric Patient: A Case Report

**DOI:** 10.7759/cureus.52963

**Published:** 2024-01-25

**Authors:** Khalaf Alnowaishiri, Hamad Alazmi, Abdulrahman Aldousari, Abdullah Alsahli, Ahlam Alharbi

**Affiliations:** 1 Orthopedic Surgery, University of Jordan, Amman, JOR; 2 Family Medicine, Primary Health Care Center, Riyadh, SAU

**Keywords:** magnetic resonance imaging, foot pain, tarsal coalition, navicular bone, cuboid bone

## Abstract

Cubonavicular coalition is a rare congenital anomaly involving fibrous or osseous fusion between the cuboid and navicular bones. This case report presents a comprehensive analysis of a 10-year-old female patient with cubonavicular coalition, detailing the diagnostic challenges and tailored therapeutic interventions. The patient presented with persistent left foot pain and restricted range of motion. Clinical examination, radiographic studies, and magnetic resonance imaging confirmed cubonavicular coalition. Laboratory investigations ruled out systemic inflammatory processes. A multidisciplinary approach was adopted, initially employing nonsteroidal anti-inflammatory drugs and physical therapy. Surgical resection of the coalition was performed due to persistent symptoms, leading to successful outcomes. This case report contributes valuable insights into the clinical presentation, diagnosis, and management of cubonavicular coalition in pediatric patients. The successful outcome underscores the importance of a comprehensive and individualized approach, providing a basis for informed decision-making in similar cases. Continued research is essential to refine therapeutic algorithms and enhance understanding of rare musculoskeletal anomalies.

## Introduction

Cubonavicular coalition, an uncommon congenital anomaly characterized by fibrous or osseous fusion between the cuboid and navicular bones, poses a diagnostic and therapeutic challenge in pediatric orthopedics. Although infrequently encountered, this condition can lead to debilitating symptoms such as foot pain and restricted range of motion [1,2]. This case report explores the presentation, diagnostic work-up, and management of a 10-year-old female patient presenting with left foot discomfort attributed to cubonavicular coalition.

While tarsal coalition in various forms has been extensively documented, cubonavicular coalition remains a distinctive entity with limited literature available. The rarity of this condition necessitates meticulous evaluation, combining clinical examination and advanced imaging modalities for accurate diagnosis. The impact of cubonavicular coalition on pediatric patients' daily activities and quality of life underscores the importance of prompt recognition and tailored intervention [2,3].

This case report aims to contribute valuable insights into the clinical manifestations, diagnostic strategies, and therapeutic interventions associated with cubonavicular coalition in the pediatric population. By presenting a comprehensive overview of the case, we seek to enhance the understanding of this unique musculoskeletal abnormality and provide a basis for informed decision-making in the management of similar cases.

## Case presentation

A 10-year-old female patient was referred to our pediatric orthopedic clinic with complaints of persistent left foot pain and limited range of motion. The symptoms had been progressively worsening over the past six months, prompting the parents to seek medical attention. The patient's medical history revealed no significant systemic illnesses or previous injuries to the affected foot. Family history was unremarkable for musculoskeletal abnormalities.

Upon physical examination, the patient demonstrated tenderness over the medial aspect of the left foot, specifically localized to the region of the naviculocuboid joint. The range of motion at the midfoot was notably restricted, with palpable stiffness during attempted inversion and eversion. No signs of inflammation or skin abnormalities were observed. Neurovascular examination of the lower extremity was within normal limits. Laboratory investigations, including complete blood count, erythrocyte sedimentation rate, and C-reactive protein, were all within normal ranges, ruling out systemic inflammatory processes (Table 1). 

**Table 1:** Comprehensive laboratory test results with reference ranges

Lab Test	Result	Reference Range
Hemoglobin	12.5 g/dL	12.0-15.5 g/dL
White Blood Cell Count	8,000 cells/mm³	4,000-11,000 cells/mm³
Platelet Count	250,000 cells/mm³	150,000-450,000 cells/mm³
Erythrocyte Sedimentation Rate	10 mm/h	0-20 mm/h
C-Reactive Protein	5 mg/L	0-10 mg/L
Rheumatoid Factor	8 IU/mL	0-14 IU/mL
Antinuclear Antibodies	Negative	Negative/Positive
HLA-B27	Negative	Negative/Positive
Serum Uric Acid	4.2 mg/dL	3.5-7.2 mg/dL
Serum Calcium	9.0 mg/dL	8.5-10.5 mg/dL
Serum Phosphorus	3.5 mg/dL	2.5-4.5 mg/dL
Serum Alkaline Phosphatase	70 IU/L	35-104 IU/L
Thyroid-Stimulating Hormone	2.5 μIU/mL	0.4-4.0 μIU/mL
Free Thyroxine	1.2 ng/dL	0.8-1.8 ng/dL
Anti-Cyclic Citrullinated Peptide	15 U/mL	0-20 U/mL
Creatinine	0.8 mg/dL	0.5-1.2 mg/dL
Blood Urea Nitrogen	15 mg/dL	8-20 mg/dL
Alanine Aminotransferase	25 IU/L	5-40 IU/L
Aspartate Aminotransferase	30 IU/L	8-40 IU/L
Alkaline Phosphatase	80 IU/L	35-104 IU/L
Sodium	140 mmol/L	135-145 mmol/L
Potassium	4.2 mmol/L	3.5-5.0 mmol/L
Chloride	100 mmol/L	98-106 mmol/L

**Figure 1:**
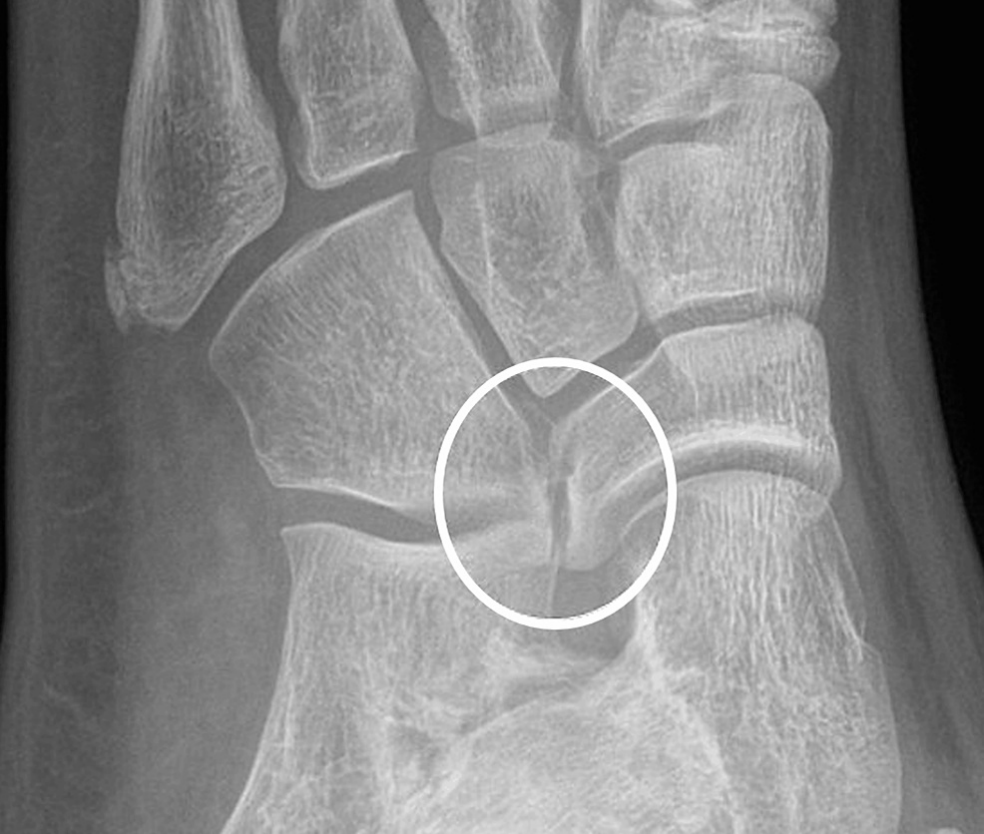
Radiograph of the left foot showing an abnormal bony connection (encircled) between the cuboid and navicular bones

**Figure 2:**
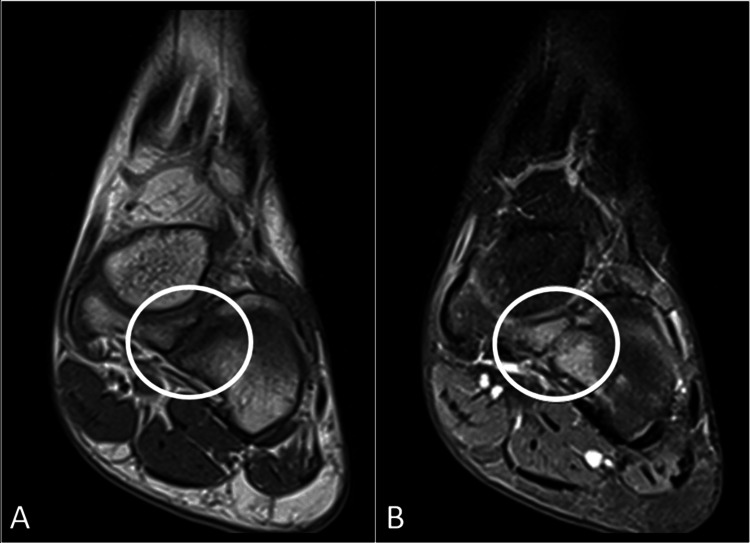
MRI of the left foot in PD-weighted (A) and T1-FS-weighted (B) images confirms the presence of fibrous fusion (encircled) at the cubonavicular joint. MRI: magnetic resonance imaging; PD: proton density, FS: fat saturated.

Given the clinical presentation, further investigations were pursued to elucidate the underlying pathology. Initial radiographic assessment of the left foot revealed an abnormal bony connection between the cuboid and navicular bones, consistent with the cubonavicular coalition (Figure 1). To characterize the extent and nature of the coalition, magnetic resonance imaging of the left foot was performed. The scan confirmed the presence of fibrous and osseous fusion at the cubonavicular joint (Figure 2).

The treatment plan included a combination of conservative measures and surgical intervention. Initially, the patient was prescribed a course of nonsteroidal anti-inflammatory drugs (NSAIDs) to alleviate pain and reduce inflammation. Physical therapy was initiated to improve joint mobility and strengthen surrounding musculature. However, due to the persistent symptoms and limited response to conservative measures, surgical intervention was deemed necessary.

**Figure 3:**
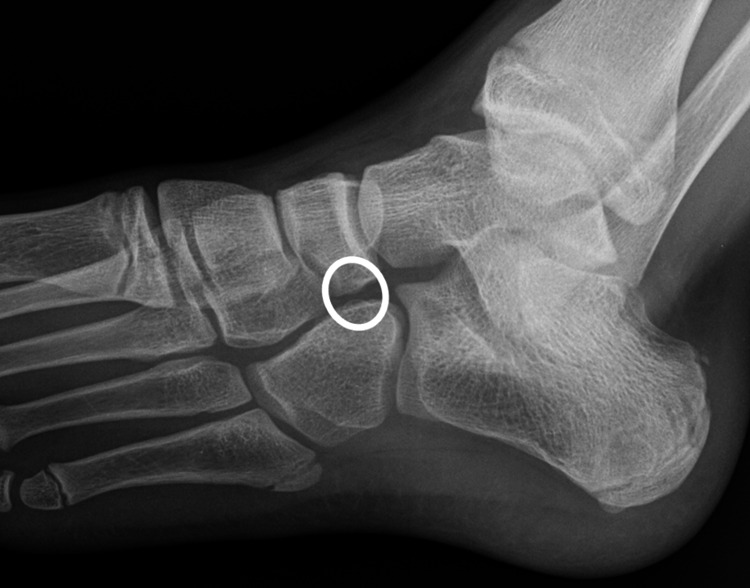
Foot radiograph shows the preserved joint space (encircled) between the cuboid and navicular bones.

The surgical procedure involved resection of the fibrous and osseous coalition, with meticulous care to preserve the adjacent joint structures. Postoperatively, the patient underwent a structured rehabilitation program to optimize functional recovery and prevent recurrence. The hospital course was uneventful, with the patient demonstrating progressive improvement in foot function and resolution of pain. Regular follow-up appointments were scheduled to monitor the patient's postoperative progress, including clinical examination and imaging studies to ensure proper healing and joint function. A repeat radiograph after 18 months revealed restoration of the normal joint space between the navicular and cuboid bones (Figure 3).

## Discussion

The presented case of a 10-year-old female with cubonavicular coalition highlights the diagnostic intricacies and therapeutic nuances associated with this rare congenital anomaly. The clinical manifestation of cubonavicular coalition is often insidious, with patients commonly presenting with nonspecific symptoms such as localized foot pain and restricted range of motion. The challenge lies in differentiating these symptoms from other more prevalent musculoskeletal conditions. In our case, the accurate diagnosis was achieved through a comprehensive approach integrating detailed clinical examination, radiographic studies, and advanced imaging with magnetic resonance imaging. This underscores the importance of a systematic and multidisciplinary assessment in cases of suspected rare musculoskeletal anomalies [3,4].

The diagnostic process should also consider the differential diagnosis, as cubonavicular coalition may mimic other conditions such as tarsal coalition or inflammatory joint disorders [2-4]. Careful exclusion of alternative diagnoses through a combination of clinical acumen and imaging findings is imperative to avoid misdiagnosis and ensure appropriate management [3,4].

Medical management in our case began with a conservative approach, employing NSAIDs to alleviate pain and reduce inflammation. Physical therapy was initiated to improve joint mobility and strengthen the surrounding musculature. While these measures form the cornerstone of initial intervention for cubonavicular coalition, the persistent nature of the symptoms and limited response to conservative measures in our patient necessitated a transition to surgical intervention.

Surgical management involved a meticulous resection of the fibrous and osseous coalition [2-5]. The goal was to optimize functional recovery and alleviate symptoms while preserving adjacent joint structures. The decision to proceed with surgery was based on the persistent nature of symptoms, aligning with existing literature that highlights the variable response to conservative measures in cases of cubonavicular coalition. The success of the surgical intervention, as evidenced by the resolution of symptoms and the restoration of foot function, underscores the importance of considering surgical options when conservative measures prove insufficient. The postoperative period included a structured rehabilitation program to ensure optimal recovery and prevent potential recurrence.

## Conclusions

This case report contributes to the limited body of literature on cubonavicular coalition, emphasizing the importance of a systematic diagnostic approach and individualized treatment strategies. The successful outcome in this case supports the efficacy of surgical intervention when conservative measures prove insufficient. Further research and collaborative efforts are warranted to accumulate additional cases, facilitating a more comprehensive understanding of cubonavicular coalition and refining therapeutic algorithms for optimal patient outcomes. The insights provided by this case underscore the significance of continued exploration into rare musculoskeletal anomalies, fostering advancements in pediatric orthopedics and enhancing clinical care for affected individuals.
